# Inhibition of Type III Interferon Expression in Intestinal Epithelial Cells—A Strategy Used by Coxsackie B Virus to Evade the Host’s Innate Immune Response at the Primary Site of Infection?

**DOI:** 10.3390/microorganisms9010105

**Published:** 2021-01-05

**Authors:** Virginia M. Stone, Emma E. Ringqvist, Pär G. Larsson, Erna Domsgen, Ulrika Holmlund, Eva Sverremark-Ekström, Malin Flodström-Tullberg

**Affiliations:** 1 Center for Infectious Medicine, Department of Medicine, Karolinska Institutet, 141 52 Stockholm, Sweden; virginia.stone@ki.se (V.M.S.); emma.ringqvist@ki.se (E.E.R.); par.larsson@cytiva.com (P.G.L.); ernadomsgen@gmail.com (E.D.); 2 Department of Molecular Biosciences, The Wenner-Gren Institute, Stockholm University, 106 91 Stockholm, Sweden; ulrika.holmlund@sll.se (U.H.); eva.sverremark@su.se (E.S.-E.); 3 Department of Research, Education and Innovation, Karolinska University Hospital, 171 64 Stockholm, Sweden

**Keywords:** Coxsackievirus (CVB), enterovirus, *IFIH1*, immune evasion, innate immunity, interferon, intestine, intestinal epithelial cells, poly I:C, type 1 diabetes

## Abstract

Increasing evidence highlights the importance of the antiviral activities of the type III interferons (IFNλs; IL-28A, IL-28B, IL29, and IFNλ4) in the intestine. However, many viruses have developed strategies to counteract these defense mechanisms by preventing the production of IFNs. Here we use infection models, a clinical virus isolate, and several molecular biology techniques to demonstrate that both type I and III IFNs induce an antiviral state and attenuate Coxsackievirus group B (CVB) replication in human intestinal epithelial cells (IECs). While treatment of IECs with a viral mimic (poly (I:C)) induced a robust expression of both type I and III IFNs, no such up-regulation was observed after CVB infection. The blunted IFN response was paralleled by a reduction in the abundance of proteins involved in the induction of interferon gene transcription, including TIR-domain-containing adapter-inducing interferon-β (TRIF), mitochondrial antiviral-signaling protein (MAVS), and the global protein translation initiator eukaryotic translation initiation factor 4G (eIF4G). Taken together, this study highlights a potent anti-Coxsackieviral effect of both type I and III IFNs in cells located at the primary site of infection. Furthermore, we show for the first time that the production of type I and III IFNs in IECs is blocked by CVBs. These findings suggest that CVBs evade the host immune response in order to successfully infect the intestine.

## 1. Introduction

The gastrointestinal tract plays a critical role in preventing infections by invading pathogens, such as viruses, however we are only just beginning to understand the details regarding how the interplay between the host, the commensal microbiota, and infecting microbes dictates permissiveness and susceptibility to infection.

Type I (α, β, ε, κ, ω) and type III (λ-1, -2, -3 and -4) interferons (IFNs) are key components of the host’s innate immune response to viruses. IFN-mediated protection against viruses is elicited through the induced expression of a number of interferon-stimulated genes (ISGs), which encode proteins that promote an anti-viral state. Type I and III IFNs elicit similar anti-viral responses. However, their target cell types vary due to differences in the expression of their cognate receptors; the type I IFN receptor is ubiquitously expressed in all nucleated cells whereas the type III IFN receptor subunits, IFNLR1 (or IL-28R) and IL-10RB (or IL-10R2), have a more restricted distribution and are found mainly in cells of epithelial origin (recently reviewed in [1,2]).

Type I IFNs protect intestinal epithelial cells (IECs) against a variety of enteric viruses and more recently, an anti-viral role of the type III IFNs has also been described. Despite the similarities between type I and III IFNs, recent studies have identified a non-redundant, sterilizing effect of the type III IFNs in the intestine against certain enteric viruses, thus highlighting a potentially important, organ specific role of these cytokines [3,4,5].

Coxsackieviruses of the group B family (CVBs), are common human single-stranded RNA viruses of the enterovirus genus. Generally, they cause either no or mild symptoms, however they have been associated with severe diseases such as myocarditis, hepatitis, meningitis, and type 1 diabetes [6,7]. CVBs infect cells through binding to the primary CVB receptor, the Coxsackievirus and adenovirus receptor (CAR), although a second receptor, namely decay-accelerating factor (DAF) is involved in CVB entry to polarized epithelial cells [6]. Currently, we have a limited understanding of what regulates susceptibility to severe CVB infections, but it is likely that both host and viral factors play a role [8,9,10]. Given that the gastrointestinal tract is the initial site of CVB infection, knowledge regarding the methods through which the intestine regulates permissiveness to infections is likely to provide further insight into possible targets for treatment and/or preventative therapeutics.

Permissiveness of IECs to CVBs has previously been documented [11,12,13] and these viruses can also persistently infect human IEC cell lines [13,14]. Intestinal epithelial organoid models with greater complexity than IEC monolayers are also permissive to CVB infection [15,16,17]. Moreover, Oikarinen et al. suggest that enteroviruses can persistently infect the human intestine and that this is more common in individuals with Type 1 diabetes compared to those without diabetes [18]. Type I IFNs protect against CVB infections in a variety of tissues [19,20,21,22] and recently a protective role of the type III IFNs against CVBs was documented in primary human islets of Langerhans [23,24] and hepatocytes [25]. Whether the type III IFNs elicit protection against CVB infections in human IECs is however currently unknown. Based upon the recently described non-redundant role of the type III IFNs against other enteric viruses [3,4], it is important to establish whether the type III IFNs have a role in controlling the replication of CVBs in cells of the intestine.

As host cells have developed methods to control viral infections, many viruses in turn have evolved sophisticated mechanisms to evade the host’s immune responses. These include targeting pathways involved in type I IFN production and signaling, modulating ISGs, preventing the cell from undergoing apoptosis, and inhibiting stress granule formation [26,27,28,29]. CVB host immune evasion strategies have been described, including the prevention of type I IFN production [30] and early cell death [27,31]. Recent studies have begun to document the scale of disruption that enterovirus encoded proteases can have on host cells through the cleavage of many cellular proteins [29,32]. It is not currently known, however, whether CVBs can also attenuate type III IFN production in IECs, as seen in our experiments conducted in HeLa cells [33].

In this study, we hypothesized that the type I and III IFNs can attenuate the replication of CVBs in human IECs, and our studies show that type III IFNs exert a potent antiviral activity against such a virus. Interestingly, we also uncovered a mechanism through which the virus may evade the host’s immune response in the intestine, namely by efficiently preventing type I and III IFN expression.

## 2. Materials and Methods

### 2.1. Virus Stocks

CVB3-V13 was originally obtained as previously described and propagated then titered in RD cells [34].

### 2.2. Cell Lines and Human Pancreatic Islets

CaCo-2 cells were kindly provided by Dr Bitte Aspenström Fagerlund, The Swedish National Food Agency, Stockholm, Sweden and HT-29 cells (HTB-38) were obtained from American Type Culture Collection (ATCC, Manassas, VA, USA). HT-29 cells were cultured in McCoys 5A medium supplemented with 10% inactivated fetal bovine serum (FBS) and CaCo-2 cells were cultured in DMEM supplemented with 20% FBS and 2 mM L-glutamine. HeLa cells were cultured in RPMI 1640 medium supplemented with 10% FBS and 2 mM L-glutamine. All cell culture media was free of antibiotics and the cells were confirmed mycoplasma negative. Cells were cultured at 37 °C, 5% CO_2_ and passaged when they reached confluency. Human pancreatic islets were isolated from a human cadaver organ donor at the Nordic Network for Clinical Islet Transplantation, Uppsala University Hospital, Sweden, and maintained as described previously [23].

### 2.3. IFN Treatment of Cells

CaCo-2 and HT-29 cells were plated in 12-well plates at 1 × 10^6^ cells/well or 1.2 × 10^6^ cells/well, respectively. Eighteen-hours post plating, IECs were mock-treated with IFN-buffer (0.1% BSA in phosphate buffer saline, PBS) or with IFNλ1 or IFNλ2 dissolved in PBS containing 0.1% BSA (both at a concentration of 100 ng/mL; Preprotech, Rocky Hill, NJ, USA) for 6 h or 24 h. IFN-α2b, also diluted in PBS-0.1% BSA, was used at a concentration of 1000 U/mL (Intron A; Merck Sharp & Dohme, Stockholm, Sweden). The concentrations of the IFNλs (100 ng/mL) and IFNα (1000 U/mL) used were based on previous studies performed in our group ([23,25] and data not shown) and from other studies that demonstrate cellular responses with no accompanying adverse effects [35]. Six or 24 h post treatment, either mRNA was isolated and used to determine changes in gene expression via real-time reverse transcription-polymerase chain reaction (RT-PCR) or protein was extracted and alterations in protein expression were studied by Western blotting. In virus infection experiments, cells were pre-treated with IFNs for 24 h, infected with CVB3 (see Section 2.4), and then cultured for 24 h with IFNs. The supernatant and cells were then collected and viral titers measured by plaque assay.

### 2.4. CVB3-V13 Infection of Cells

A number of pilot experiments (data not shown) were carried out in order to optimize infection conditions. Cells were plated in 24 well plates at a concentration of 4 × 10^5^ cells/well (CaCo-2) or 5 × 10^5^ cells/well (HT-29) and cultured for 96 h. At this time point, separate wells to those used for infection were used to calculate the average number of cells/well by manual counting. Cells were then washed with PBS and either mock infected (serum free media alone) or infected with CVB3-V13 diluted to the indicated multiplicity of infection (MOI) in their respective serum free medium for 1 h. During this period, the plates were rocked every 10 min. After 1 h infection media was removed, the cells were washed 3 times with PBS (the last wash was saved) and then incubated for the indicated time points in complete media. If IFNλ1, IFNλ2, or IFNα were present in the culture medium prior to infection, they were re-added to the culture medium at the same concentrations for the remainder of the experiment. After infection, cells and/or supernatant were harvested at the specified time points for determining viral titers (standard plaque assay), gene expression analysis (RT-PCR), and protein changes (Western blotting). Plaque forming units (PFUs/mL) were calculated as a measure of viral titers (replicating virus) in the cells and supernatant using a standard plaque assay in HeLa cells [22].

To ensure that no virus was present after the extensive washing stages, the last wash was saved and titers of replicating virus determined by standard plaque assay in HeLa cells. Titers of replicating virus present in the final wash (an indication of virus present at 0 h) were deducted from the titer measurement determined at 24 h in the cells and culture supernatant. This data is presented as the log_10_ PFU/mL. Furthermore, the PFU/mL at the point of infection was also calculated (PFU = MOI × no. of cells) and compared to the PFU of samples collected at 24 h p.i. to confirm to the virus successfully replicated in IECs.

### 2.5. RNA Extraction and qRT-PCR

Total RNA was extracted from CaCo-2 and HT-29 cells using the RNeasy mini kit (Qiagen, Sollentuna, Sweden). RNA concentration and purity were measured using a NanoDrop ND-1000 spectrophotometer (Saveen Werner AB, Limhamn, Sweden). First, 1.5 μg of total RNA was treated with DNase then converted to cDNA using the Superscript III First Strand synthesis system (Invitrogen, Paisley, UK) according to the manufacturer’s instructions. Commercially available primers (Quantitech Primer Assay, Qiagen, Sollentuna, Sweden) were used in combination with RT^2^ real-time™ SYBR Green/ROX Polymerase chain reaction (PCR) master mix (Qiagen, Sollentuna, Sweden) to quantify the mRNA expression of IFNLR1, IL-10RB, IFNλ1, IFNλ2, PKR, OAS-2, MxA, ISG15, iNOS, TLR3, MDA5, RIG-I, and glyceraldehyde 3-phosphate dehydrogenase (GAPDH). The same cDNA was used to examine IFNβ using commercially available Taqman primers and the Taqman mastermix (Thermo Fisher Scientific, Stockholm, Sweden). Real-time PCR was performed using an ABI Prism 7500 Sequence detecting system (Thermo Fisher Scientific, Stockholm, Sweden). Gene expression levels were normalized to the expression of GAPDH (Ct). The data are presented as 2^−ΔCt^. In all experiments, a Ct value of ≥35 was considered to be below the detection level and thus genes with a Ct value of greater than 35 were deemed to be not expressed.

### 2.6. Western Blotting and Antibodies

Cellular extracts from IFN-treated or CVB3-V13 infected cells were extracted and Western blotting performed as described previously [23]. Primary antibodies against MxA (1:1000; kindly provided by Professor Otto Haller, University of Freiburg, Germany), MAVS/IPS-1 (1:1000; Enzo Life Sciences, Lausen, Switzerland), TRIF (1:1000; Cell Signalling, Leiden, The Netherlands), eIF4G (1:1000; Cell Signalling), and β-actin (1:10,000, MP Biomedicals, Aurora, OH, USA) were incubated overnight with the membrane at 4 °C in 5% BSA in PBS. Binding of primary antibody was detected using an HRP-conjugated anti-rabbit or anti-mouse antibody (1:3000; both Bio-Rad, Sundbyberg, Sweden). Blots were developed using Supersignal West Dura extended-duration substrate (Pierce, Älvsjö, Sweden) and a Fujifilm LAS-4000 imaging system (GE Healthcare Bio-Sciences AB, Uppsala, Sweden). For Western blots of PKR and ISG15 SuperSignal West Femto Maximum Sensitivity Substrate by Pierce was used (Fisher Scientific, Göteborg, Sweden) and the blots were detected by ChemiDoc XRS+ analyser (Bio-Rad, Sundbyberg, Sweden) with the Image Lab Software (Bio-Rad). Primary antibodies against PKR (1:1000, Cell Signalling, Leiden, The Netherlands), ISG-15 (1:750, Santa Cruz Biotechnology, Heidelberg, Germany) and β-actin (here diluted 1:30,000) were incubated overnight in TBS-T at 4 °C in 5% FCS. Binding of primary antibodies to PKR, ISG15, and actin were detected using an HRP-conjugated anti-rabbit (1:100,000, ThermoFisher Scientific, Göteborg, Sweden) or anti-mouse antibody (1:120 from 10 µg/mL, Pierce, Älvsjö, Sweden).

### 2.7. Flow Cytometry

Cell surface expression of CAR and DAF were evaluated using flow cytometry. Binding of primary antibodies against CAR (clone RmcB, Millipore, Solna, Sweden) and DAF (biotinylated clone 143-30, eBioscience, Frankfurt, Germany) were detected using a secondary rat-anti-mouse antibody with a FITC conjugate (BD Pharmingen, Stockholm, Sweden) and Streptavidin-conjugated APC (eBioscience, Frankfurt, Germany), respectively. HeLa cells were used as positive control cells for both CAR and DAF expression. IL-10Rβ and IFNλR1 (IL-28Rα) expression were detected by directly labelled AF488 and PE antibodies, respectively (Clone 90,220 and Clone 601,106, R&D Systems, Oxon, UK) on cells negative for Live/Dead Fixable Far Red Dead Cell Stain (Fisher Scientific, Göteborg, Sweden). Isotype controls with directly labelled IgG1,κ were purchased from the same vendor as the primary antibodies. Intracellular staining was performed using the BD Cytofix/Cytoperm Fixation/Permeablization Kit as described by manufacturer (BD Biosciences, San Jose, CA, USA). All flow cytometry experiments were carried out on a BD Accuri C6 flow cytometer and data was analyzed using FlowJo (TreeStar Inc., Ashland, OR, USA; version 9-10.7.1).

### 2.8. Poly (I:C) Treatment

IECs were plated at a concentration of 4 × 10^5^ cells/well (CaCo-2) or 5 × 10^5^ cells/well (HT-29) and cultured for 96 h then treated with vehicle (water alone), 10 ng/m or 30 ng/mL poly (I:C) (Sigma-Aldrich, Stockholm, Sweden) for 3 h or 6 h, and RNA extraction was performed as previously described.

### 2.9. Poly (I:C) Transfection

CaCo-2 and HT-29 cells were plated at a concentration of 2 × 10^5^ cells/well in a 24-well plate. Twenty-four hours after plating, cells were transfected with 10 ng/mL poly (I:C) or 6 μg/mL pmaxGFP™ (a transfection positive control, Lonza, Stockholm, Sweden) using lipofectamine 2000 (Life Technologies, Stockholm, Sweden) according to the manufacturer’s instructions. RNA was extracted after 3 h or 6 h (as previously described) or cells transfected with pmaxGFP™ were cultured for 24 h and the transfection efficiency was determined by flow cytometry (data not shown).

### 2.10. Statistical Analysis

All statistical analysis was performed the Graphpad Prism 5 software (Graphpad Software, La Jolla, CA, USA). Multiple comparisons were performed using a One-way ANOVA with Bonferroni correction and single comparisons were made using the Student’s *t*-test with Welch’s correction. A *p*-value <0.05 was considered statistically significant. Data is presented as the mean ± standard deviation (S.D.).

## 3. Results

### 3.1. CaCo-2 and HT-29 Cells Are Permissive to CVB3 Infection

**Figure 1:**
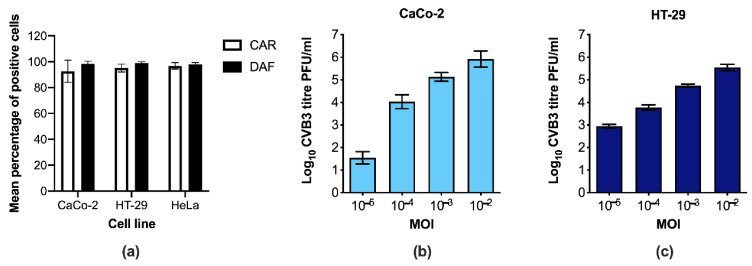
Intestinal epithelial cells (IECs) express Coxsackievirus and adenovirus receptor (CAR) and decay-accelerating factor (DAF) and can be infected by Coxsackievirus B3 (CVB3) in a dose-dependent manner. (**a**) The expression levels of the enterovirus receptors Coxsackie and adenovirus (CAR) and decay-accelerating factor (DAF) were assessed in CaCo-2 and HT-29 IECs by flow cytometry. At least 50,000 cells were screened in each experiment (CaCo-2 *n* = 7, HT-29 *n* = 7, HeLa *n* = 4). Shown are means ± SD. (**b**) CaCo-2 and (**c**) HT-29 cells were infected with 10^−5^ to 10^−2^ multiplicity of infection (MOI) CVB3 for 24 h and virus replication in the cells and supernatant was measured 24 h p.i. by standard plaque assay. Data are presented as mean plaque forming unites (PFUs) ± SD from three independent experiments per cell line. Significant increases in virus titers were observed in all pair-wise comparisons (*p* < 0.05, one-way ANOVA).

To start with, we confirmed that two commonly used model systems for human IECs, the cell lines CaCo-2 and HT-29, express CAR and DAF as shown in Figure 1a. Subsequently, we investigated whether the cells can be productively infected with CVBs by using different concentrations of Coxsackievirus B3 (CVB3) and measuring titers of replicating virus in cells and supernatants at 24 h post infection (p.i.). No replicating virus was detected in the mock-infected control cells (data not shown). In contrast, there was a clear dose response increase in the titers of replicating virus in both cell lines with increasing concentrations of virus (CaCo-2 cells Figure 1b and HT-29 cells Figure 1c). Furthermore, the amount of replicating virus particles in both cell lines was approximately 100–1000 times higher 24 h p.i. when compared to the infectious dose at 0 h p.i., confirming that the virus successfully replicated in both IECs (data not shown). Taken together, these results indicate that human IECs are permissive to infection with CVB3.

### 3.2. Human IECs Enter an Antiviral State after Type I and III IFN Treatment

**Figure 2:**
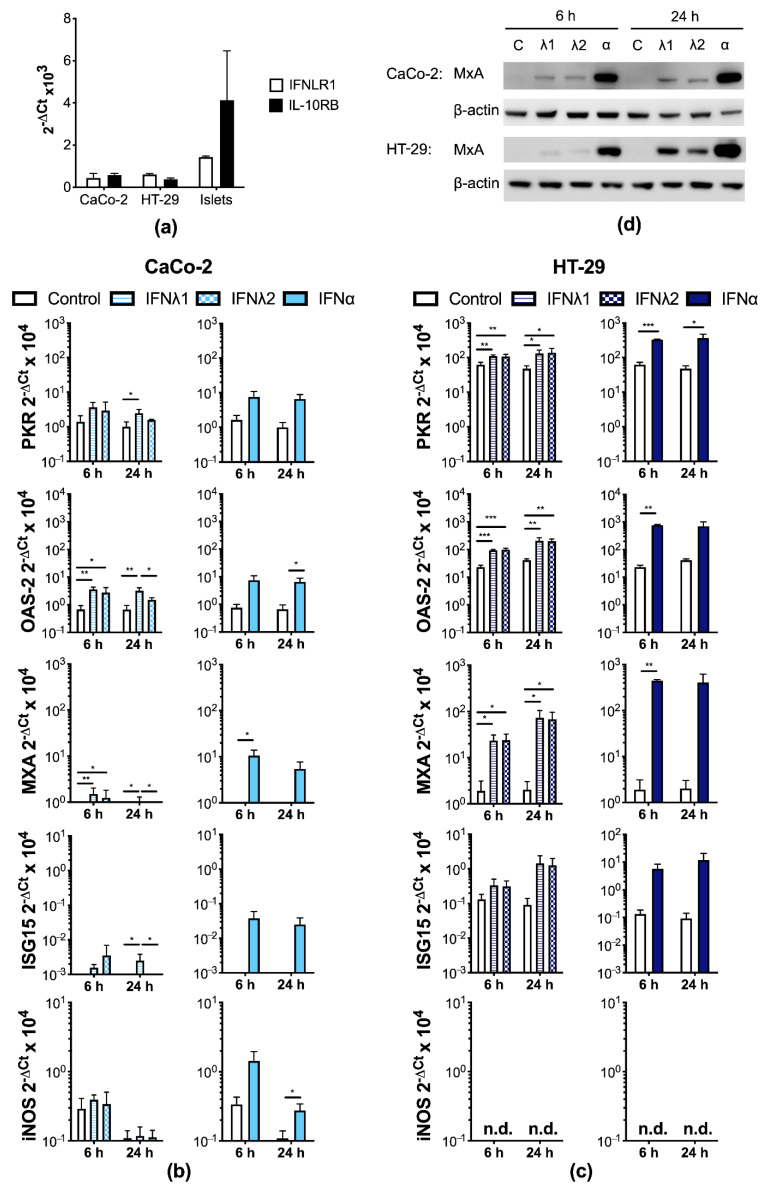
Intestinal epithelial cells (IECs) respond to Type I and III interferons (IFNs) through the up-regulation of interferon stimulated genes (ISGs) at the mRNA and protein level. (**a**) IFNλ receptor subunits mRNA (IFNLR1/IL-28Rα and IL-10RB) were detected by real-time PCR in IECs and human pancreatic islets (a positive control; n = 2 donors). Data are presented as the means ± SD from three independent experiments per cell line. (**b**–**d**) IECs were treated with vehicle, IFNλ1, IFNλ2 (both at concentration of 100 ng/mL), or IFNα (1000 U/mL) for 6 h or 24 h and then RNA (**b**,**c**) or protein (**d**) were extracted. (**b**,**c**) The expression of ISGs (protein kinase regulated by dsRNA, PKR; 2′,5′-oligoadenylate synthetase 2, OAS-2; myxovirus resistance protein 1, MXA; interferon-induced 17 kDa protein, ISG15; and inducible nitric oxide synthase, iNOS) were measured by real-time PCR in (**b**) CaCo-2 and (**c**) HT-29 cells. Data are presented as the mean ± SD from at least three independent experiments per cell line. * *p* < 0.05, ** *p* < 0.01, *** *p* < 0.001 as determined by one-way ANOVA or Student’s *t*-test. n.d., none detected. (**d**) Myxovirus resistance protein 1 (MxA) protein levels were examined by Western blot. Representative image from three experiments (all with similar results).

We next determined whether the type III IFNs trigger a biological response in IECs. To assess whether IECs express the type III IFN receptor we measured the mRNA expression of the two subunits. Both cell lines expressed mRNA encoding IFNLR1 and IL-10RB (Figure 2a; cDNA from human pancreatic islets from one donor was used as a positive control; [23]). Furthermore, we performed FACS analysis examining the protein expression of the two receptor subunits and found both subunits were detected in HT-29 and CaCo-2 cells (Supplementary Figure S1a).

Following this, we investigated whether IECs enter an antiviral state after treatment with the type III IFNs through the examination of a number of ISGs. We included type I IFN (IFNα) as a positive control. Treatment of CaCo-2 cells with either IFNλ1 or IFNλ2 increased the expression of protein kinase regulated by dsRNA (PKR), 2′,5′-oligoadenylate synthetase 2 (OAS-2), myxovirus resistance protein 1 (MxA), and interferon-induced 17 kDa protein (ISG15; Figure 2b). Significant increases in PKR mRNA expression were seen after 24 h of treatment with IFNλ1, in OAS2 mRNA expression after 6 h and 24 h of treatment with IFNλ1 and 6 h treatment with IFNλ2, and in MXA mRNA expression after 6 h and 24 h of treatment with IFNλ1 and 6 h treatment with IFNλ2. In contrast, there were no differences in inducible nitric oxide synthase (iNOS) expression in CaCo-2 cells after treatment with either of the type III IFNs used. In a similar manner to the type III IFNs, IFNα treatment increased PKR, OAS-2, and MXA mRNA expression in CaCo-2 cells and significant increases were found for OAS-2 and MxA (Figure 2b). Moreover, IFNα mediated increases in iNOS expression were also detected in CaCo-2 cells (Figure 2b).

In a similar manner to the CaCo-2 cells, HT-29 cells also significantly up-regulated the expression of PKR, MxA, and OAS-2 after treatment with either IFNλ1, IFNλ2, or IFNα (Figure 2c). The expression of ISG15 in HT-29 cells also increased although these values did not reach statistical significance (Figure 2c). In contrast to CaCo-2 cells, HT-29 cells did not express iNOS at the mRNA level after treatment with any of the IFNs (Figure 2c).

We next performed Western blotting to determine whether the changes observed in mRNA expression following IFN treatment were reproducible at the protein level. As shown in Figure 2d, IFNλ1/2 and IFNα treatment increased the expression of MxA in HT-29 and CaCo-2 cells. Moreover, we confirmed that CaCo-2 cells express PKR and ISG-15 at the protein level and there appeared to be a slight up-regulation of both of these genes after IFNλ1/2 treatment after 24 h treatment (Supplementary Figure S1b). These results are similar to the mRNA data (Figure 2b). Collectively, these results indicate that human IECs enter an antiviral state following treatment with the type I and III IFNs through the up-regulated expression of a number of ISGs.

### 3.3. CVB3 Replication Is Perturbed in Human IECs Treated with Type I and III IFNs

**Figure 3:**
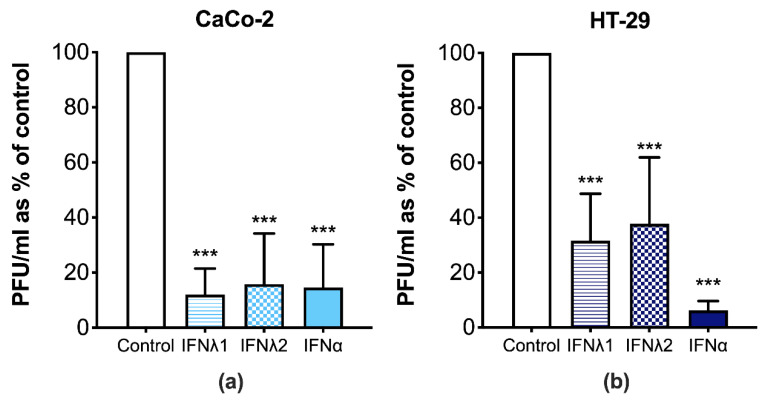
Type I and III interferons (IFNs) protect against Coxsackievirus B3 (CVB3) infection in intestinal epithelial cells (IECs). (**a**) CaCo-2 and (**b**) HT-29 cells were treated with vehicle, IFNλ1, IFNλ2 (both at concentration of 100 ng/mL), or IFNα (1000 U/mL) for 24 h and were then infected with CVB3 at a multiplicity of infection (MOI) of 10^−4^ for 24 h. Virus titers in cells and supernatants were measured 24 h p.i. by standard plaque assay. Data are presented as the mean plaque forming units (PFUs) ± SD from at least four independent experiments per cell line. Significant decreases in virus titers were observed in all treatments when compared to the vehicle alone (*** *p* < 0.001; one-way ANOVA).

Next, we investigated whether the IFN-induced antiviral response can attenuate the replication of CVB3 in IECs. Both type I and III IFNs significantly reduced CVB3 replication when compared to the mock-treated CVB3 infected cells, as seen by a decrease in the viral titers (Figure 3). These results suggest that the type I and III IFNs are capable of attenuating CVB3 replication in IECs.

### 3.4. Human IECs Do Not Up-Regulate the Expression of Type I and III IFNs in Response to CVB3 Infection

**Figure 4:**
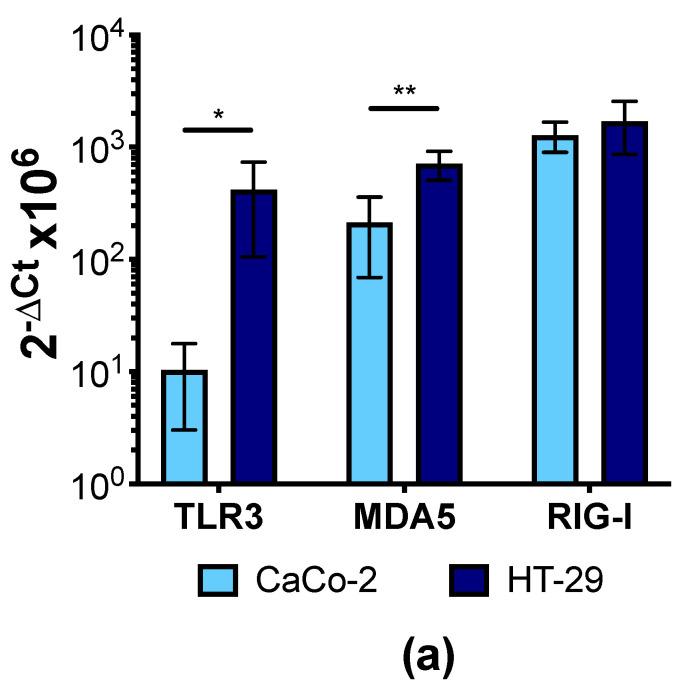

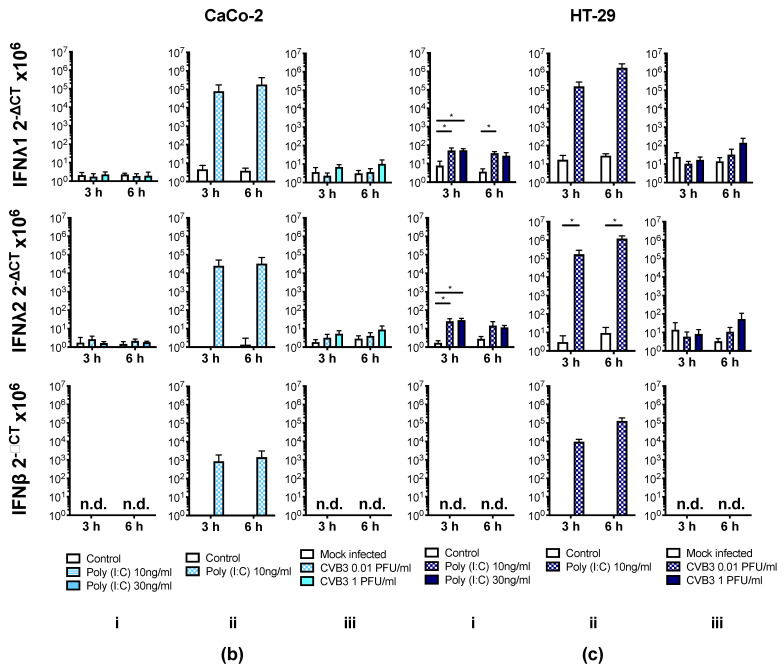
Polyinosinic:polycytidylic acid (poly (I:C)) transfection, but not Coxsackievirus B3 (CVB3) infection, induces the expression of interferons (IFNs) in intestinal epithelial cells (IECs). (**a**) The expression of the pattern recognition receptors toll-like receptor 3 (TLR3), melanoma differentiation-associated protein 5 (MDA-5), and retinoic acid-inducible gene 1 (RIG-I) were confirmed in CaCo-2 and HT-29 cells at the mRNA level by real-time PCR. (**b**) CaCo-2 or (**c**) HT-29 cells were treated with 10 ng/mL or 30 ng/mL poly (I:C) (i) or transfected with 10 ng/mL poly (I:C) (ii) or infected with 0.01 or 1 plaque forming units (PFU)/mL CVB3 (iii) for 3 h or 6 h. The expression of the type I or III IFNs at the mRNA level was measured by real-time PCR. Data presented as the mean ± SD from at least three independent experiments per cell line (* *p* < 0.05 and ** *p* < 0.01; Student’s *t*-test with Welch’s correction or one-way ANOVA).

Having shown that IFNλ1/2 and IFNα attenuate CVB3 replication in human IECs, we next investigated whether human IECs express IFNs in response to CVB3 infection. Initially, we verified that the signaling pathways required for the sensing of CVBs and induction of type I and III IFNs are present and function in IECs. CVBs are recognized primarily by the pattern recognition receptor (PRR) melanoma differentiation associated protein 5 (MDA5), but can also be recognized by toll-like receptor 3 (TLR3; [9]). Both of these receptors as well as retinoic acid-inducible gene 1 (RIG-I) were expressed at the mRNA level in CaCo-2 and HT-29 cells (Figure 4a). As these PRRs have been implicated in the detection of the viral dsRNA mimic poly (I:C) [36], we next treated or transfected the cells with poly (I:C) to assess whether the pathways leading to the transcription of type I and III IFNs are intact in CaCo-2 and HT-29 cells. Poly (I:C) added directly to the tissue culture media is expected to be taken up and sensed by TLR3, and this treatment led to a small but significant up-regulation of IFNλ1 and IFNλ2 mRNA in HT-29 cells, but no significant increase in CaCo-2 cells (Figure 4(ci,bi), respectively). Furthermore, neither cell line upregulated the type I IFN, IFNβ after poly (I:C) treatment (Figure 4(bi,ci)). In contrast, when we transfected IECs with poly (I:C), a method to deliver the dsRNA directly to the cytosol allowing for recognition by MDA5/RIG-1, there were large increases in the expression of both IFNλ1 and IFNλ2 and also IFNβ at the mRNA level in both cell lines (Figure 4(bii,4cii)), thus indicating that the pathways required for the induction of type I and III IFN expression were functional.

We next infected CaCo-2 and HT-29 cells with CVB3 and examined the mRNA expression of the type I and III IFNs at 3 h and 6 h post infection (p.i.). Both cell lines showed a trend towards a small increase in IFNλ1 and IFNλ2 mRNA expression, which, however, failed to reach statistical significance (Figure 4(biii,ciii)). Neither cell line expressed IFNβ (Figure 4(biii,ciii)). From these results, we show that both CaCo-2 and HT-29 cells up-regulate the expression of type I and III IFNs after delivery of dsRNA to the cytosol, and they are also up-regulated in HT-29 cells following treatment with exogenous poly (I:C). However, these increases are not observed when the IECs are infected with CVB3.

### 3.5. CVB3 Infection Results in the Cleavage of Proteins Involved in IFN Production

**Figure 5:**
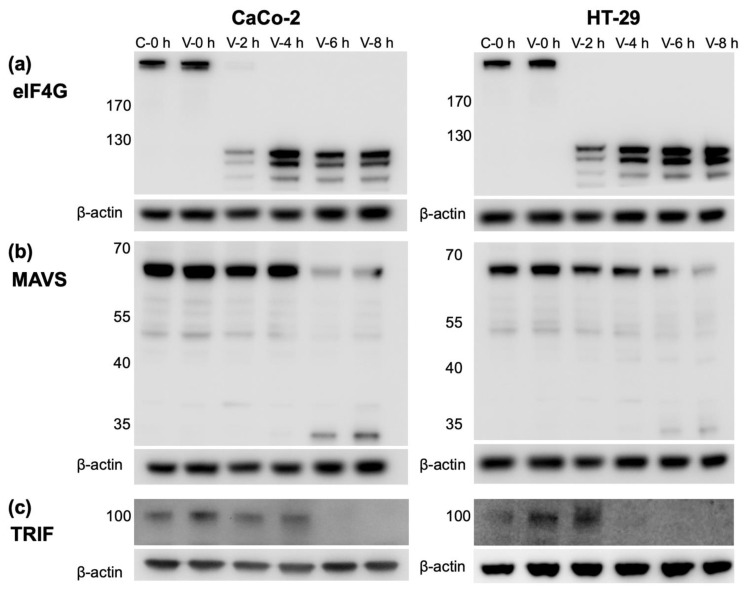
Coxsackievirus B3 (CVB3) interferes with proteins involved IFN production. (**a**–**c**) Intestinal epithelial cells were mock infected (C-0 h) or infected with CVB3 (multiplicity of infection, MOI = 1; V-0 h–V-8 h) and protein was extracted at 0, 2, 4, 6, and 8 h p.i. The presence of eukaryotic initiation factor 4G (eIF4G; **a**), mitochondrial antiviral signaling protein (MAVS; **b**) and TIR-domain-containing adaptor-inducing interferon-β (TRIF; **c**) were detected by Western blot and β-actin was used as a loading control. Representative images from a minimum of two experiments with similar results.

CVBs have been shown to evade the host immune response through the cleavage of proteins involved the induction of type I IFNs [37]. Therefore, we investigated whether the proteolytic cleavage of key proteins involved in IFN production occurs in IECs after CVB3 infection. Eukaryotic initiation factor 4G (eIF4G) is involved in the initiation of protein translation and is cleaved by some viruses (including CVBs) to aid the translation of their own genome and to prevent host translation [38,39]. CVB3 degraded eIF4G in both CaCo-2 and HT-29 cells around 2 h p.i. and there was a complete loss of eIF4G protein expression up to 8 h p.i. (Figure 5a), indicating that the virus impedes host global protein translation. A potential cleavage product was detected from 2 h p.i. in both cell lines (although it has not been sequenced to fully confirm its identity).

The viral mRNA sensors MDA5 and RIG-1 signal through the mitochondrial antiviral-signaling protein (MAVS), whilst TLR3 conveys its signals via TRIF, although both pathways converge after these molecules and initiate type I and III IFN production [9]. We next examined whether the expression of the downstream signaling proteins MAVS and TRIF are altered after CVB3 infection in IECs [38,39,40,41]. The protein level of MAVS began to decrease at around 4–6 h p.i. in both CaCo-2 and HT-29 cells, which correlated with the appearance of a possible cleavage product of approximately 35 kDa in size (Figure 5b). In a similar manner to MAVS, TRIF protein had disappeared by 6 h post CVB3 infection in CaCo-2 cells and by 4 h in HT-29 cells and was also absent at 8 h post p.i. (Figure 5c). These results suggest that CVB3 cleaves the proteins eIF4G, MAVS, and TRIF in IECs, which are involved in global protein translation (eIF4G) and interferon production (MAVS and TRIF).

## 4. Discussion

The present study highlights a function of the type III IFNs in preventing CVB infection in IECs. Furthermore, we identify a strategy employed by CVB3 to evade the immune response in cells present at the primary site of infection. By preventing type III IFN production in IECs, the virus may favor its own replication and dissemination, and possibly the establishment of persistent infections in the gastrointestinal tract.

Exposure of CaCo-2 and HT-29 cells to type I or III IFNs induced the expression of several ISGs, which have been previously described to play an important role in the defense against CVBs, including MxA [42], ISG15 [43], the RNase L and OAS-2 pathway [44], and PKR [44]. The increased expression of these genes was paralleled by an attenuation in CVB3 replication. Although the exact mechanism(s) through which permissiveness to infection is reduced are a focus for future studies, it is clear that it/they may occur through the aforementioned proteins. iNOS, which also has an important function in the host antiviral defense against systemic CVB infection [45,46], was not required for type I and III IFN mediated protection in IECs, as neither cell type showed significant increases in iNOS expression after treatment with the IFNs.

In certain tissues, CVB infection initiates the expression of the type III IFNs [23,24,25]. Similarly, other viruses, such as MCMV [35] and rotavirus [47], have been shown to induce the expression of type I and III IFNs in IECs. In stark contrast to these observations, we discovered that CVB3 infection led to no IFNβ expression and only low, yet non-significant increases in IFNλ1 and IFNλ2 expression. We found that the lack of response was not due to a general failure of the IECs to respond to infection, as both CaCo-2 and HT-29 expressed the PRRs shown to recognize CVBs (MDA5/RIG-I and TLR3; [9]) and responded to the cytosolic delivery of the viral dsRNA mimic poly (I:C) by inducing the expression of both type I and III IFNs.

Our results suggest IECs cannot robustly respond to CVB3 infection through the production of IFNs. This lack of response led us to wonder whether CVBs have the capacity to interfere with the ability of IECs to produce IFNs. Many viruses, including CVBs, have developed strategies to evade the host’s immune response in order to favor their own replication, including blocking type I and III IFN production and/or signaling [26,27,28,29,30,31]. It has been suggested that the CVB encoded proteases 2A^pro^ and 3C^pro^ are involved in the degradation of proteins involved in virus recognition and the induction of a type I IFN response [30,41]. Moreover, we recently uncovered an important role for the viral protease 2A^pro^ in preventing CVB3 induced type III IFN production in HeLa cells [33]. Here, we discovered that CVB3 infection in IECs leads to a reduced expression of intact MAVS and TRIF, key proteins involved in IFN production. Both proteins decreased in expression between 4 h and 6 h post CVB3 infection and a potential cleavage product appeared in both of the IEC blots probed for MAVS, although we did not sequence this product to confirm it is indeed a fragment of MAVS. No band that could respond to a possible cleavage product was seen with TRIF. These results are in line with those documented by Mukherjee et al. [41] and Feng et al. [30] with regards to type I IFN production and our own studies examining the type III IFNs [33]. We also show that eIF4G, a key factor in the global protein translation is also degraded by CVB3 in IECs, and blockage of protein translation may further prevent IFN production [38]. Taken together, our study shows that CVB3 not only attenuates the induction of the type I IFNs but also the type III IFNs. To the best of our knowledge, this is the first time this loss of IFNλ transcript production (alongside the loss of the type I IFNs) has been documented in IECs infected with an enterovirus.

The precise relevance of the decreased type I and III IFN signaling in the intestine after CVB infection remains to be established, however numerous studies have indicated the significant role of the type III IFNs in the gastrointestinal tract. A few years ago, Pott et al. [48] reported the importance of the type III IFNs in controlling rotavirus infection in IECs in vivo in mice, where the type I IFNs were unable to compensate for a loss in IFNλ signaling. Baldridge [49] and Nice [50] have also described the key role that IFNλs have in controlling persistent murine norovirus infections. More recent studies by Nice et al. [50] demonstrated that exogenous IFNλ administration can clear persistent and acute norovirus infections in a manner that is independent of the adaptive immune response. Another pertinent and very recent study reports the importance of the type I and III IFNs in protecting against SARS-CoV-2 replication in human IECs [51]. Furthermore, the studies by Baldridge et al. report a potential link between the host’s microbiota and the antiviral response determined by the IFNλs [49]. A fifth study, performed by Mahlakõiv et al. [52], suggests, using a murine reovirus infection model, that there is a compartmentalized IFN system in the gut mucosa with the epithelial cells responding predominantly to the IFNλs, whereas the remainder of the tissue relies on the type I IFNs in the defense against viruses. In contrast to our studies, where we show that human IEC lines respond well to IFNα, they suggest that IECs do not respond to the type I IFNs due to low levels of expression of the type I IFN receptor in these cells. These variations may have arisen due to differences between human and murine systems or the in vivo versus in vitro systems used. Regardless of this, it is without doubt that the IFNλs play an irreplaceable role in control of enteric infections in the intestine, and also this most likely encompasses infections with the CVBs.

It is thought that enteroviruses are able to persist in the human intestine due to the presence of virus in the feces a long time after the acute infection has been cleared [53]. This aids the efficient spread of the virus from host to host. Our results describing the ability of CVBs to block type III IFN production in IECs may give an indication of the mechanisms employed by the virus to establish persistence in the intestine and enable further spread.

Enterovirus infections have been associated with the initiation of processes that lead to the development of type 1 diabetes [7]. Prolonged shedding of enterovirus in the feces was recently linked to an increased risk of developing pancreatic islet autoantibodies in genetically predisposed children [54]. The underlying causes of this long-term viral shedding are yet to be determined, but in view of this observation, it is of interest that one of our previous studies linked a type 1 diabetes-associated polymorphism in the gene encoding MDA5, *IFIH1* (rs199760), to a poor type III IFN response to CVB3 infection [24]. Our current study points towards the idea that the type III IFNs have an important role in protecting the intestine from unrestrained enterovirus replication. This coupled with studies by others showing that type III IFNs are uniquely responsible for the control of early and persistent infections by other enteric viruses (e.g., [50]), raise the possibility that the type III IFNs play a crucial role in regulating enterovirus persistence in the human gut. Moreover, they suggest that this defense pathway may be weaker in individuals at risk of developing type 1 diabetes, leading to prolonged viral shedding. These ideas require further verification in future studies.

Finally, an additional observation made in this study that requires some discussion, is the result showing that poly (I:C) transfection led to a large up-regulation in the expression of IFNλ1, -λ2, and IFNβ in both CaCo-2 and HT-29 cells. In contrast, poly (I:C) treatment resulted in small significant increases in IFNλ expression in HT-29, but not CaCo-2 cells. Furthermore, IFNβ mRNA was not detected in either cell line. The discrepancy between poly (I:C) transfection and treatment may be due to the non-phagocytic nature of IECs as suggested by Hirata et al. [55], which prevents poly (I:C) binding to TLR3 in its endosomal location. Intracellular receptors such as MDA5 and RIG-I may be key in the detection of cytosolic dsRNA including poly(I:C) delivered via transfection. Other studies have suggested that IECs, including HT-29 and CaCo-2 cells can respond to longer poly(I:C) exposure than those used in the present study (6 h) [56]. Based on the aforementioned and our present observations it is possible to hypothesize that IECs are slightly more inert to exposure with PAMPs and less likely to respond to dsRNA (e.g., poly (I:C)) to avoid the continual induction of an inflammatory state in the gut.

## 5. Conclusions

In summary, our studies demonstrate that the type I and III IFNs can attenuate CVB3 infection in human IECs. Mounting evidence suggests that the IFNλs fulfil an important, non-redundant role in the intestine in the protection against viruses. This may account for the evasion strategies employed by CVBs that attenuate IFN production through the prevention of IFN gene expression. These findings provide important new insights into the complicated interactions between CVBs, type I and III IFNs, and IECs and suggest that the administration of exogenous IFNλs could be beneficial in the prevention of acute CVB infections, and in turn may perhaps cure persistent CVB infections. Our study collectively proposes an important role for both the type I and III IFNs in the protection against CVBs in the intestinal epithelium.

## Data Availability

The data presented in this study is available from the corresponding author upon reasonable request.

## Supplementary Materials

The following are available online at https://www.mdpi.com/2076-2607/9/1/105/s1, Figure S1: IECs express the IFNλ receptor subunits at the protein level and respond to IFNλ treatment by increasing the protein levels of PKR and ISG15.
