# Supplementary material for: Inhibition of Type III Interferon Expression in Intestinal Epithelial Cells—A Strategy Used by Coxsackie B Virus to Evade the Host’s Innate Immune Response at the Primary Site of Infection?
Source: Microorganisms. 2021 Jan 5;9(1):105. doi: 10.3390/microorganisms9010105 (PMC7824802; doi:10.3390/microorganisms9010105)
Supplement: Supplementary file 1 [file microorganisms-09-00105-s001.pdf]

microorganisms

Supplementary Information

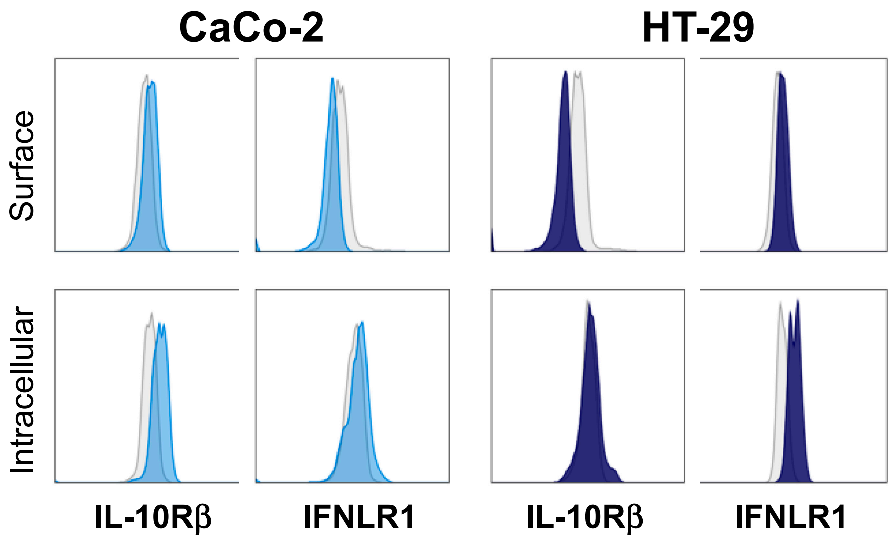

(a)

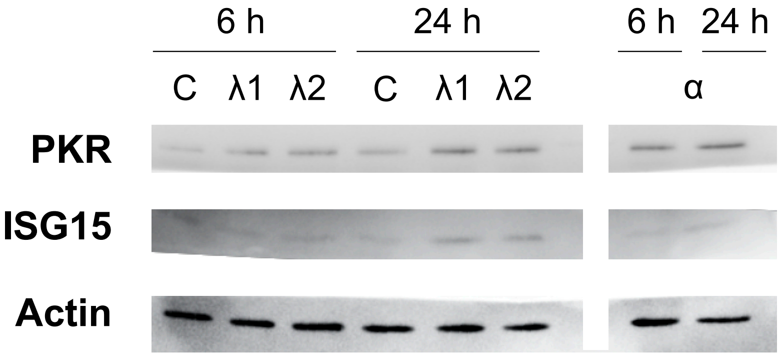

(b)

Supplementary Figure S1: IECs express the IFNλ receptor subunits at the protein level and respond to IFNλ treatment by increasing the protein levels of PKR and ISG15. (**a**) IFN-lambda receptors subunits, IL-10Rβ and IFNLR1, were detected on CaCo-2 (light blue) and HT-29 cells (dark blue) by flow cytometry. Surface staining is depicted on top row, intracellular levels in the same cells is depicted on bottom row. Filled histograms indicate modal expression of IL-10Rβ or IFNLR1, corresponding isotype controls are shown in light grey. The result of one out of 1-2 experiments with similar results are shown. (**b**) SDS-PAGE followed by Western blot detection of the proteins PKR and ISG15 in CaCo-2 cells after IFN-treatment for 6 or 24 hours. 1μg protein was loaded per sample. Actin was used as loading control, and all three proteins were detected on the same membrane, which were cut in three pieces before incubation with primary antibody followed by a HRP-conjugated secondary antibody. The Western blot shown is representative of two independent experiments with similar results.
